# Nutrition after preterm birth and adult neurocognitive outcomes

**DOI:** 10.1371/journal.pone.0185632

**Published:** 2017-09-28

**Authors:** Sara Sammallahti, Eero Kajantie, Hanna-Maria Matinolli, Riikka Pyhälä, Jari Lahti, Kati Heinonen, Marius Lahti, Anu-Katriina Pesonen, Johan G. Eriksson, Petteri Hovi, Anna-Liisa Järvenpää, Sture Andersson, Katri Raikkonen

**Affiliations:** 1 Department of Psychology and Logopedics, University of Helsinki, Helsinki, Finland; 2 Children’s Hospital, Helsinki University Hospital and University of Helsinki, Helsinki, Finland; 3 National Institute for Health and Welfare, Helsinki, Finland; 4 PEDEGO Research Unit, MRC Oulu, Oulu University Hospital and University of Oulu, Oulu, Finland; 5 Helsinki Collegium for Advanced Studies, Helsinki, Finland; 6 University BHF Centre for Cardiovascular Sciences, Queen’s Medical Research Institute, University of Edinburgh, Edinburgh, United Kingdom; 7 Folkhälsan Research Center, Helsinki, Finland; 8 Department of General Practice and Primary Health Care, University of Helsinki and Helsinki University Hospital, Helsinki, Finland; 9 Vasa Central Hospital, Vasa, Finland; Centre Hospitalier Universitaire Vaudois, FRANCE

## Abstract

**Background:**

Preterm birth (<37 gestational weeks) poses a risk of poorer neurocognitive functioning. Faster growth after preterm birth predicts better cognitive abilities and can be promoted through adequate nutrition, but it remains unknown whether variations in nutrient intakes translate into long-term benefits for neurodevelopment.

**Methods:**

In 86 participants of the Helsinki Study of Very Low Birth Weight Adults (birthweight <1500g), we examined if higher intakes of energy, macronutrients, and human milk during the first nine weeks after preterm birth predict performance in tests of cognitive ability at 25.1 years of age (SD = 2.1).

**Results:**

10 kcal/kg/day higher total energy intake at 3 to 6 weeks of age was associated with 0.21 SD higher adult IQ (95% Confidence Interval [CI] 0.07–0.35). Higher carbohydrate and fat intake at 3–6 weeks, and higher energy intake from human milk at 3–6 and at 6–9 weeks were also associated with higher adult IQ: these effect sizes ranged from 0.09 SD (95% CI 0.01–0.18) to 0.34 SD (0.14–0.54) higher IQ, per one gram/kg/day more carbohydrate and fat, and per 10 kcal/kg/day more energy from human milk. Adjustment for neonatal complications attenuated the associations: intraventricular hemorrhage, in particular, was associated with both poorer nutrition and poorer IQ.

**Conclusion:**

In preterm neonates with very low birth weight, higher energy and human milk intake predict better neurocognitive abilities in adulthood. To understand the determinants of these infants' neurocognitive outcome, it seems important to take into account the role of postnatal nutrition, not just as an isolated exposure, but as a potential mediator between neonatal illness and long-term neurodevelopment.

## Introduction

Preterm birth (before 37 gestational weeks) poses a risk of poorer neurocognitive functioning. This risk is highest for those born very preterm (<32 weeks) or at very low birth weight (VLBW; <1500g), and effects span beyond childhood. [1–4] Explaining this vulnerability are factors that underlie and result from preterm birth, including pregnancy complications and immaturity-related health problems. All these factors can reflect on slow neonatal growth, which predicts poorer adult neurocognitive outcome. [5,6] However, it remains unknown whether variations in nutrient intakes after preterm birth not only accelerate growth, but also translate into long-term benefits for neurodevelopment.

In preterm children, higher neonatal energy intake predicts better neurodevelopment, but neonatal illness may underlie this association. [7] We know of only a handful of studies that have examined nutrition after preterm birth in relation to neurodevelopmental outcomes beyond childhood, and these studies have not specifically addressed the issue of neonatal complications potentially influencing both nutrition and neurodevelopment. One set of studies included preterm individuals with birth weight <1850g, who were originally recruited into two separate randomized feeding trials and whose data were later combined. Within this population, those who received multinutrient-enriched preterm formula, versus non-fortified donor milk or standard formula, had higher verbal intelligence quotient (VIQ) at 13–20 years of age. [8,9] In a subset of the same population, those who received more maternal milk had higher VIQ at 13–20 years. [10] Contrastingly, another study of small-for-gestational-age VLBW adults found no association between neonatal energy intake and general intelligence at 23 years of age. [11]

In our cohort of adults born preterm at VLBW, we examined whether intakes of energy, carbohydrates, protein, fats, and human milk during the first nine weeks of life predict general intelligence, memory, attention, and executive functioning at 25 years of age, when taking into account important pregnancy-related and neonatal factors.

## Methods

### Participants

The *Helsinki Study of Very Low Birth Weight Adults* has been previously described. [12,3] We invited the 255 individuals born between 1978 and 1985 with VLBW who were discharged from the neonatal intensive care unit of Children's Hospital at Helsinki University Central Hospital, Finland, the only such unit in the Uusimaa province, and who lived in the greater Helsinki area as adults, to participate in the first clinical visit in 2004–2005: 166 participated. In 2007–2008, 159 of these 166 individuals could be traced and invited to a second visit, which included neurocognitive testing: 113 participated. [3,6,13–15] We excluded participants with blindness (n = 2), cerebral palsy (n = 6), or intellectual developmental disability (n = 1), and those for whom sufficient nutrition data were not available (n = 18), resulting in an analytic sample of 86 participants (mean gestational age 29 weeks, standard deviation [SD] = 16 days, range 24–35 completed weeks; mean birth weight 1116g, SD = 219g, range 600-1480g; mean age at follow-up 25.1 years, SD = 2.1 years, range 21.6–29.7 years). All participants gave written informed consent, and the Ethics Committee for Children and Adolescents’ Diseases and Psychiatry at Helsinki University Central Hospital approved the study protocol.

### Selective attrition

We compared the analytic sample (n = 86) with those who could not be included because of non-participation or missing data ("drop-outs", n = 145), and found no differences in gestational age, sex, birth weight standard deviation score (SDS), parental education, maternal smoking during pregnancy, preeclampsia, neonatal complications, nutrition in infancy, age at follow-up, or neurocognitive outcomes (p-values>0.05). We excluded all those with blindness (n = 2), cerebral palsy (n = 16), or intellectual developmental disability (n = 6) from these comparisons. The number of drop-outs whom we could include in each of the comparisons varied (gestational age, sex, birth weight, and preeclampsia data were available for all 145 drop-outs, maternal smoking data for 137 drop-outs, parental education for 80 drop-outs, neonatal complication data for 105–145 drop-outs, nutritional data for 37–53 drop-outs, age at follow-up for 18 drop-outs, and neurocognitive data for 16–17 drop-outs).

### Nutrition

Nutritional data during the initial hospital stay came from hospital records and was available for the first 9 weeks of life (after which the number of participants with sufficient data was reduced because of hospital discharge). As previously described in more detail, [16] we divided the data into 3-week periods (birth to 3, 3–6, and 6–9 weeks of age), and calculated daily mean *total energy intakes* and *energy intakes from protein*, *fat*, *and carbohydrates* from all enteral and parenteral nutrition, and *energy intake from human milk*, including donated and mother's own milk, per kilogram bodyweight. The macronutrient content of the mother's own milk was estimated based on the nutritional composition data published by Anderson et al. [17], who followed the milk content of mothers who delivered preterm. The nutritional composition of banked human donor milk was based on values published by Rönnholm et al. [18,19], who analyzed the macronutrient content of the banked milk used in the hospital where the infants of the present study were treated.

Enteral feeding was initiated through a nasogastric tube with human milk on 1^st^-2^nd^ day of life. Milk intake was increased to a maximum of 200ml/kg/day according to individual tolerance, and maintained at this level until discharge. All milk was pasteurized. During the 9-week period, 59 participants (69%) received mother's own milk, 81 (94%) received pooled donor milk, and 19 (22%) received formula. If targeted enteral feeding was not possible, intravenous fluids with glucose were initiated, and amino acids and lipids were gradually introduced from the 2^nd^-3^rd^ day.

### Neurocognitive outcomes

We used four subtests of the Wechsler Adult Intelligence Scale-III [20] (Vocabulary, Digit span, Similarities, Block design) to estimate *full-scale*, *verbal*, *and performance intelligence quotient* (IQ, VIQ, and PIQ, respectively). As measures of executive functioning, attention, and visual memory, we used Phonetic (words beginning with letters S and P) and Categorical (animal, vegetable/fruit names) Verbal Fluency [21], the Rey-Osterrieth Complex Figure Test [22], the Trail Making Test [23], the Bohnen version of the Stroop Test [24], and the Conners’ Continuous Performance Test [25]. Indices of these latter five tests correlate highly, and we thus used principal components analysis with Varimax rotation for data reduction. The first four components with eigenvalues >1 explained 75% of the total variation [6] and were named *Verbal flexibility* (higher scores reflected better Fluency and Stroop scores), *Visual flexibility* (better Trail Making Test scores), *Visual memory* (better Rey-Osterrieth Complex Figure test scores), and *Impulsivity* (faster Conners’ Continuous Performance Test reaction times and more commission errors) (S1 Table).

### Confounders

From medical and birth records, we collected *sex*, date of birth for calculating *age at adulthood follow-up*, self-reported *maternal smoking during pregnancy* (no/yes), *preeclampsia* (no/yes, diagnosed using standard criteria [26]) *gestational age* based on last menstrual period and confirmed by a neonatologist (ALJ), and birth weight for calculating *birth weight SDS* according to Finnish standards. [27] During the adulthood follow-up, participants reported the *highest education of either parent* (basic/secondary/lower tertiary/upper tertiary), as proxy of parental neurocognitive abilities and socio-economic status. As *neonatal complications*, collected from medical records, we included *septicemia* (no/yes, diagnosed if infant showed symptoms and a blood culture was positive), *bronchopulmonary dysplasia* (no/yes, diagnosed by a neonatologist [ALJ] based on Northway criteria [28]), *patent ductus arteriosus* treated with indomethacin (no/yes, including those who also underwent corrective surgery), *blood exchange transfusion* due to hyperbilirubinemia (no/yes), duration of *ventilator treatment* (no/0-7/8-14/15-28/>28 days), and *intraventricular hemorrhage* (IVH) (no/grade I-II/grade III-IV). No participant was diagnosed with necrotizing enterocolitis. Neonatal cerebral ultrasound was being introduced during the study period, [29] and despite limited equipment and personnel, only 22 participants lacked data on IVH. Four participants lacked data on maternal smoking during pregnancy. These were dummy-coded into separate groups. Two participants who lacked all neonatal complication data were excluded in the analyses which adjusted for these complications.

### Statistical analysis

We used linear regression models to test, first, if total energy intake and energy intake from human milk from birth to 3, 3 to 6, and 6 to 9 weeks of age predicted IQ, PIQ, VIQ, Verbal flexibility, Visual flexibility, Visual memory, and Impulsivity. Moreover, we tested associations between protein, fat and carbohydrate intake (g/kg/day) during the same three-week periods, and IQ. We square-transformed IQ and PIQ to improve linear model fitting, and standardized the outcomes within the sample (mean = 0, SD = 1) to facilitate comparison of effect sizes.

In Model I, we adjusted for gestational age, sex, birth weight SDS, and age at follow-up. In Model II, we adjusted for Model I factors, parental education, maternal smoking during pregnancy, and preeclampsia. In Model III, we adjusted for Model I factors and the neonatal complications described above. We also tested if associations varied by sex or by birth weight SDS, by including a product term (‘sex x nutrition’ or ‘birth weight SDS x nutrition’) into the regression equation accompanied by main effects and other Model I covariates. Further, we tested whether total energy intakes, energy intakes from human milk, and IQ differed between those with versus those without each neonatal complication, and whether IQ differed between those who received mother's own milk versus those who did not: we used analysis of variance for duration of ventilation treatment and t-tests for dichotomized complications and for feeding with mother's own milk. We used IBM SPSS Statistics 24 for statistical analyses and considered two-tailed p-values<0.05 statistically significant.

## Results

Characteristics, nutritional intakes in infancy, and neurocognitive test scores in adulthood are presented in Table 1.

**Table 1:** Background characteristics, nutritional intakes in infancy, and adult IQ, VIQ, and PIQ among very low birth weight (<1500g) adults.

*Characteristic*	*M (SD)*	*n (%)*	*Participants*
**Background characteristics**			
Gestational age, weeks	29.0 (2.2)		86
Gestational age <32 weeks		80 (93)	86
Gestational age <28 weeks		25 (29)	86
Sex, male		37 (43)	86
Birth weight, g	1116 (219)		86
Birth weight, SD score	-1.2 (1.5)		86
Birth weight ≤-2 SD		27 (31)	86
Birth weight <1000 grams		26 (30)	86
Birth length, cm	37 (2.4)		85
Birth head circumference, cm	26 (2.0)		83
Mother smoked during pregnancy		16 (20)	82
Preeclampsia		17 (20)	86 ^a^
Age at clinical follow-up visit, years	25.1 (2.1)		86
Highest education of a parent			86
basic/primary or less		8 (9)	
upper secondary		16 (19)	
lower tertiary		35 (41)	
upper tertiary		27 (31)	
**Neonatal complications and illnesses** ^b^			
Duration of ventilator treatment, median days (25^th^ to 75^th^ percentile / range)	6 (0–21 / 0–80)		84
Septicemia		8 (10)	84
Bronchopulmonary dysplasia		22 (26)	84
Patent ductus arteriosus treated with indomethacin		29 (35)	84
Blood exchange transfusion		14 (17)	84
Intraventricular hemorrhage			64
none		50 (78)	
grade I or II		10 (16)	
grade III or IV		4 (6)	
**Mean nutrient intakes in infancy**			
Birth to three weeks of age			
Energy, kcal/kg/day	94 (17)		86
Energy from human milk, kcal/kg/day	77 (24)		83
Carbohydrates, g/kg/day	11 (1.4)		86
Protein, g/kg/day	1.4 (0.4)		86
Fats, g/kg/day	4.3 (1.2)		86
Three to six weeks of age			
Energy, kcal/kg/day	119 (15)		82
Energy from human milk, kcal/kg/day	108 (22)		78
Carbohydrates, g/kg/day	12 (1.3)		82
Protein, g/kg/day	1.9 (0.4)		82
Fats, g/kg/day	5.9 (1.0)		82
Six to nine weeks of age			
Energy, kcal/kg/day	125 (15)		79
Energy from human milk, kcal/kg/day	108 (26)		75
Carbohydrates, g/kg/day	13 (1.4)		79
Protein, g/kg/day	2.1 (0.5)		79
Fats, g/kg/day	6.2 (1.0)		79
**Estimated general intelligence scores**			
IQ, standardized score	102 (16)		86
VIQ, standardized score	104 (14)		86
PIQ, standardized score	99 (20)		86

^a^ We compared mothers among whom we had confirmed diagnosis of preeclampsia to mothers with no indication of preeclampsia in hospital or maternity clinic records.

^b^ 84 participants had data available on the duration of ventilation treatment, septicemia, bronchopulmonary dysplasia, patent ductus arteriosus for which indomethacin was given, and blood exchange transfusion, and were thus included in the analyses where we adjusted for neonatal complications.

Abbreviations: g: gram; IQ: full-scale intelligence quotient; kcal: kilocalorie; M: mean; n: number of participants in described category; Participants: number of participants for whom data were available; PIQ: performance intelligence quotient; SD: standard deviation; VIQ: verbal intelligence quotient

### General intelligence, total energy intake, and energy intake from human milk

**Fig 1:**
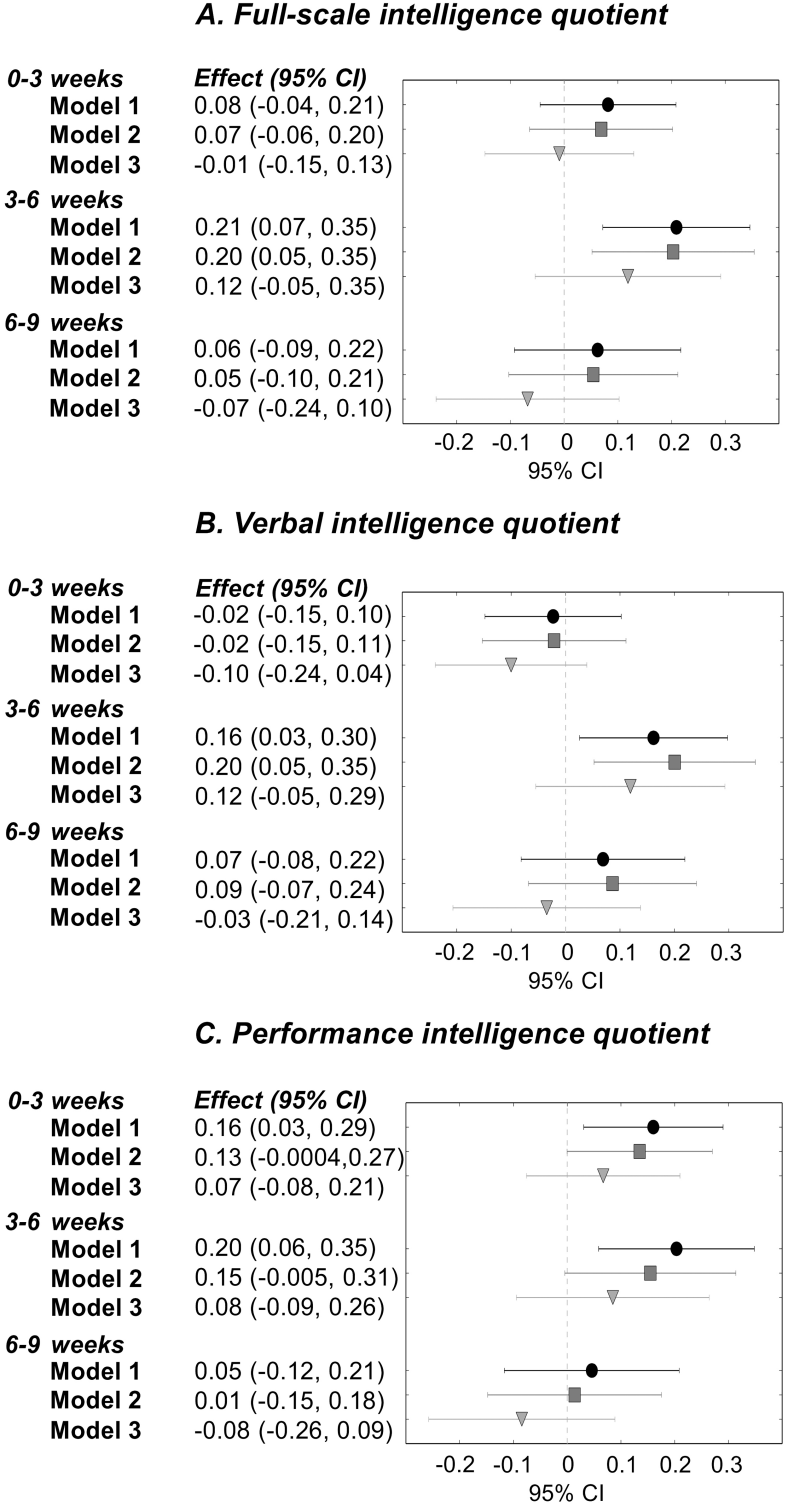
Total energy intake from birth to 3 weeks, 3 to 6 weeks, and 6 to 9 weeks of age, and full-scale, verbal and performance intelligence quotient in young adulthood, among individuals born with very low birth weight (<1500 grams).^a^ Model I: adjusted for gestational age, sex, birth weight standard deviation score, and age at follow-up. Model II: adjusted for Model I factors, parental education, maternal smoking during pregnancy, and preeclampsia. Model III: adjusted for Model I factors and neonatal complications, including septicemia, bronchopulmonary dysplasia, patent ductus arteriosus, blood exchange transfusion, duration of ventilator treatment, and intraventricular hemorrhage. ^a^ The number of participants in each analysis varied according to data availability. At 0–3 weeks, 86 participants; at 3–6 weeks, 82 participants; and at 6–9 weeks, 79 participants had data available on total energy intake and were included in the analyses (Model 1–2). In Model 3, we had to further exclude two people because of missing data on neonatal complications. Abbreviations: CI: confidence interval; Effect: change in full-scale, verbal, and performance intelligence quotient scores, in SD units, for each 10kcal/kg/day increase in total energy intake.

**Fig 2:**
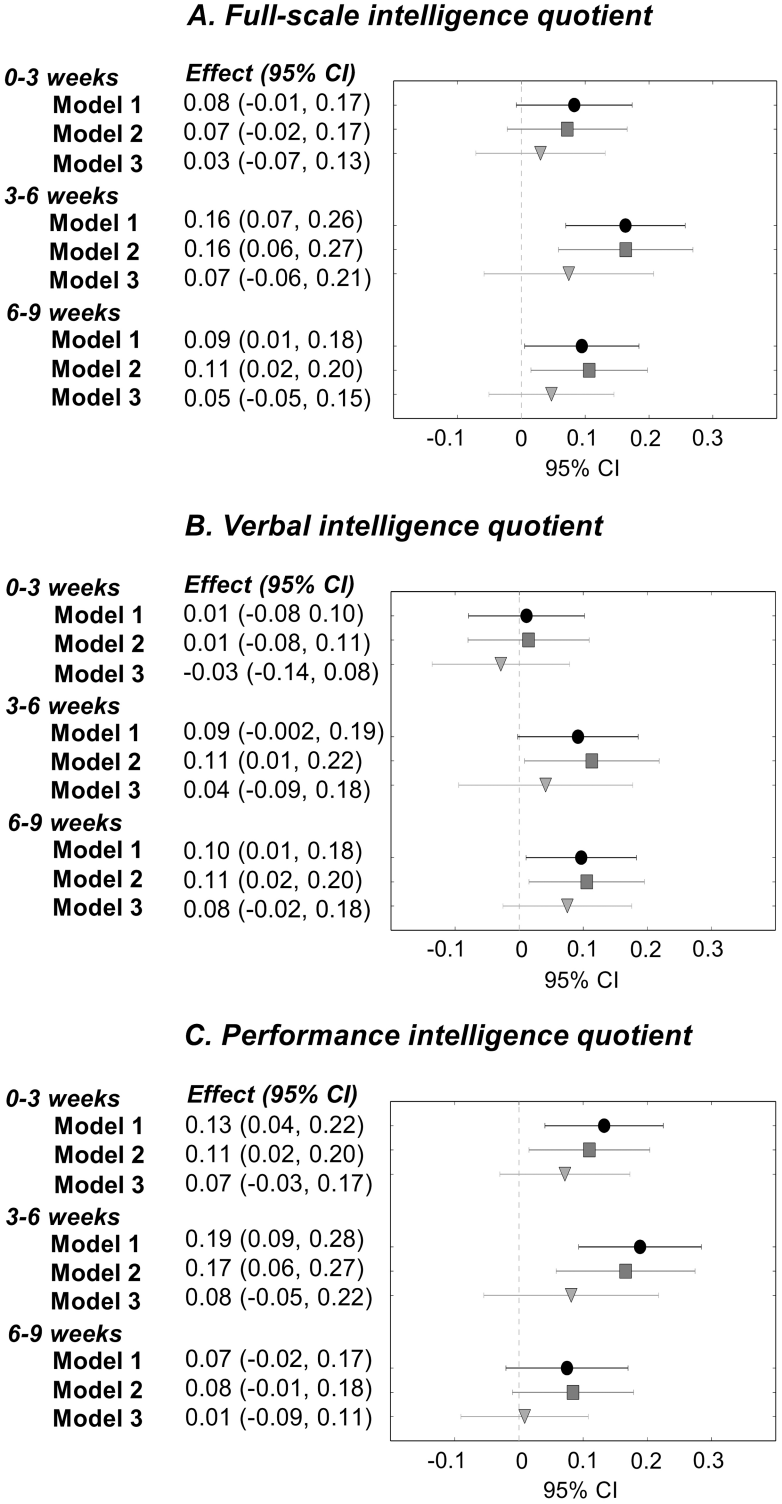
Energy intake from human milk from birth to 3 weeks, 3 to 6 weeks, and 6 to 9 weeks of age, and full-scale, verbal and performance intelligence quotient in young adulthood, among individuals born with very low birth weight (<1500 grams).^a^ Model I: adjusted for gestational age, sex, birth weight standard deviation score, and age at follow-up. Model II: adjusted for Model I factors, parental education, maternal smoking during pregnancy, and preeclampsia. Model III: adjusted for Model I factors and neonatal complications, including septicemia, bronchopulmonary dysplasia, patent ductus arteriosus, blood exchange transfusion, duration of ventilator treatment, and intraventricular hemorrhage. ^a^ The number of participants in each analysis varied according to data availability. At 0–3 weeks, 83 participants; at 3–6 weeks, 78 participants; and at 6–9 weeks, 75 participants had data available on human milk intake and were included in the analyses (Model 1–2). In Model 3, we had to further exclude two people because of missing data on neonatal complications. Abbreviations: CI: confidence interval; Effect: change in full-scale, verbal, and performance intelligence quotient scores, in SD units, for each 10kcal/kg/day increase in energy intake from human milk.

Fig 1 presents associations between total energy intake from birth to 3, 3–6, and 6–9 weeks and general intelligence: Those with higher total energy intake from birth to 3 weeks had higher PIQ (0.16 SD per 10kcal/kg/day), and those with higher total energy intake at 3–6 weeks had higher IQ, VIQ, and PIQ (0.16–0.21 SD per 10kcal/kg/day) (Model I). Fig 2 presents associations between energy intake from human milk and general intelligence: Those who received more energy from human milk from birth to 3 weeks had higher PIQ (0.13 SD per 10kcal/kg/day), those with higher milk intake at 3–6 weeks had higher IQ and PIQ (0.16–0.19 SD per 10kcal/kg/day), and those with higher milk intake at 6–9 weeks had higher IQ and VIQ (0.09–0.10 SD per 10kcal/kg/day) (Model I).

Associations remained similar after further adjustment for parental education, maternal smoking during pregnancy, and preeclampsia (Model II), but adjustment for neonatal complications attenuated them, rendering them non-significant (Model III) (Figs 1 and 2).

### General intelligence and carbohydrate, protein, and fat intakes, and the intake of mother's own milk

Higher carbohydrate intakes at 3–6 weeks were associated with higher IQ and VIQ; higher protein intakes from birth to 3 weeks with higher PIQ, and higher fat intakes between birth and 6 weeks with higher IQ, VIQ and PIQ in Model I (effect sizes 0.18–0.73 SD, per gram/kg/day of carbohydrates/protein/fat) (S2 Table). Only the associations between fat intakes at 3–6 weeks and IQ and VIQ survived adjustment for neonatal complications (Model III, effect size 0.26 for both IQ and VIQ, p-values<0.05).

In an additional analysis, we compared those who received mother's own milk (n = 59) against those who did not (n = 27): no statistically significant differences in IQ, VIQ, or PIQ between the two groups were observed (p>0.07).

### Attention, memory and executive functioning, total energy intake, and energy intake from human milk

Better Visual memory was predicted by higher total energy intake from birth to 3 weeks (effect size 0.19 SD per 10kcal/kg/day more energy, 95% CI 0.05–0.32 in Model I, p-value = 0.02 in Models II and III) and at 3–6 weeks (effect 0.22 SD; 95% CI 0.06–0.38 in Model I, p-values = 0.01 and 0.35 in Models II and III, respectively), and higher energy intake from human milk from birth to 3 weeks (effect 0.11 SD per 10kcal/kg/day more energy from human milk, 95% CI 0.01–0.21 in Model I, p-values = 0.06 and 0.08 in Models II and III, respectively). We found no other statistically significant associations between total energy intake or energy intake from human milk, and Visual memory, Verbal or Visual flexibility, or Impulsivity (p-values>0.11, Model I).

### Differences according to neonatal complications, sex, and birth weight SDS

As most associations attenuated after adjustment for neonatal complications, we specified their effects by testing associations between neonatal complications, nutrient intakes, and IQ. Those with IVH had lower total energy intake, lower energy intake from human milk, and lower IQ (p-values<0.04) (S3 Table). Those with longer ventilator treatment, bronchopulmonary dysplasia, and patent ductus arteriosus also had lower total energy intake and lower energy intake from human milk (p-values<0.03); although mean IQ's were slightly lower in those with these complications, compared with those without the complications, the differences were not statistically significant (p-values>0.12).

Associations between total energy/human milk/carbohydrate/protein/fat intakes and IQ did not vary according to birth weight SDS or to sex (p-values>0.06 for interactions).

## Discussion

In this well-characterized cohort of 86 VLBW individuals, we show that higher energy and human milk intakes during the initial hospital stay were associated with better cognitive functioning in adulthood. The effect sizes of these associations are quite consistent with earlier reports of childhood outcomes [30]: 10 kcal/kg/day higher total energy intake at 3 to 6 weeks was associated with approximately 3 point higher IQ at 25 years of age (equivalent of 0.21 SD). As compared with this period of 3 to 6 weeks, associations between nutrition from birth to 3 weeks or from 6 to 9 weeks and adult IQ were similar in direction, but smaller in effect size. The association between energy intake at 3 to 6 weeks and neurocognitive outcomes appeared to be due to energy from human milk, carbohydrates, and fat. By contrast, protein intake slightly earlier, from birth to 3 weeks, seemed to predict neurodevelopment, particularly non-verbal performance. Overall, early nutrition predicted outcomes ranging from verbal and visuospatial functioning to memory, but it did not predict executive functioning scores.

The associations we found between energy, human milk, carbohydrate, protein, and fat intakes and neurodevelopment were independent of sex, gestational age, intrauterine growth, maternal smoking, preeclampsia, and parental socio-economic status. Neither were they explained by manifest developmental disability nor neurosensory impairment.

Interestingly, when a range of neonatal complications were taken into account, no independent associations between nutrition and adult IQ remained, as demonstrated by the reduced effect sizes. IVH, which seemed to be the main down-driver of the effect sizes, was associated with both lower nutritional intakes and poorer IQ. These findings may suggest that nutritional differences reflected, or possibly mediated, the severity of neonatal illness and its effect on neurodevelopment.

Our adult findings are in line with previous observational studies in ELBW [7,30] / VLBW [31] children, which have demonstrated that higher energy, [7,30] protein, [30] and lipid [31] intakes during the first weeks of life predict better neurodevelopmental scores at 12–22 months, but these effects are driven by the most critically ill neonates. [7] Also in line with our results, randomized controlled studies and meta-analyses have failed to show that multinutrient-enriched, [32,33] protein/amino acid enriched [34–36] or long-chain poly-unsaturated fatty acid-enriched [37,38] (enteral [32–38] / parenteral [33,35,36]) nutrition during initial hospitalization after preterm birth would improve neurodevelopmental scores at or before 12 months, [33,34,37] between 18–24 months, [32,34–37] or in school-age. [34,35,38]

The most compelling evidence exists for breast milk, which predicts better neurodevelopmental outcomes in preterm children at 2–11 years [39–43]–effects that seem less evident at 9–20 months [44,45] and may be diminished when adjusting for maternal intelligence. [46] In our population, who routinely received human milk, neonatal complications seemed to largely explain the association between better neurodevelopment and higher milk intake during the first nine weeks of life, and we did not find differences in IQ between the majority of participants who received mother's own milk during the initial hospitalization and the minority who did not. However, the benefits of breastfeeding were beyond the scope of this study and any far-reaching conclusions concerning the use of mother's own milk based on these data would be ill-advised.

We are aware of only a handful of studies on nutrition after preterm birth which have spanned beyond childhood. In agreement with our results, energy intake during the first 10 days of life was not associated with IQ at 23 years in 46 small-for-gestational-age individuals born with VLBW in 1967–1975. [11] In that study, neonatal complications were not addressed—however, the VLBW newborns' survival rate of 39% at the time suggests that only the healthiest cohort members could be followed-up. [11] In another set of studies, in preterm individuals with birth weight <1850g, those randomized to receive multinutrient-enriched formula (vs. term-formula or donor milk) [8,9] and those who non-randomly received more maternal milk [10] had higher VIQ at 13–20 years of age. The positive effects contrast with the findings in younger children: even within that same population, fortified formula did not consistently improve neurodevelopmental scores at previous follow-ups at 18 months [47] and 7.5–8 years [48]. While it could be that some effects of early nutrition only become apparent with age, attrition complicates interpretation of the findings: of the total of 926 participants who were originally enrolled in those trials, data for only 95 [8], 76 [9], and 50 [10] participants were available at 13–20 years. Further, data on neonatal complications were not available, making it thus difficult to determine the extent to which our findings agree with those results.

Our study strengths include the long follow-up of VLBW individuals to adulthood, validated and extensive outcome data, and detailed pre- and postnatal data. The neonatal nutrition data recorded by medical staff is exceptional for such a long follow-up. In an era when neonatal cerebral ultrasound was just being introduced, 64 of our 86 study participants underwent the scan: a strength for an adult follow-up, yet a limitation in comparison to modern-day cohorts where cerebral ultrasound has widely become routine practice.

Our main study limitation is attrition: 113 VLBW individuals participated in the clinical visit (71% of those invited), and after excluding those with neurosensory impairments and missing nutritional data, we had an analytic sample of 86 individuals. Although those whom we could not include because of missing data did not differ from the analytic sample, loss of follow-up may cause selection bias and impact the generalizability of the results, especially into less healthy groups. We have refrained from post-hoc power calculations, [49] but note that the relatively small number of participants may hinder the detection of small-scale effects, and did not permit us to study subgroup-specific effects, such as potential mediating effects of nutrition within the most severely ill of the VLBW individuals. Neither could we examine how the timing of neonatal complications affected the outcomes, or compare the use of mother's own milk specifically against donor milk or formula use, for example.

Another limitation of our study is that although the volumes of mother's own milk were recorded in detail, we did not have data available on any inter-individual or intra-individual variation in milk composition, and thus estimated the nutrient content of maternal milk based on previous research on lactating mothers of preterm infants.[17] Further, our participants, born in 1978–1985, may not be representative of preterm infants born in high-income settings today: pre- and postnatal care have improved and the rates of IVH, for example, have declined. [50] The mean total energy, carbohydrate, and fat intakes all fell below currently recommended levels (of 110-135kcal, 11.6–13.2g, and 4.8–6.6g/kg/day, respectively) during the first 3-week period, and mean protein intakes were well below current recommendations (3.5–4.5 g/kg/day) for the entire 9-week period. [51] The risk of residual confounding always remains. Finally, a small proportion of infants received nutritional products for which we were unable to trace the exact compositions: for those products, we used the composition data of a closely corresponding product (for example, the same label with composition information available for the previous year). [16]

## Conclusions

Our study suggests that the intake of macronutrients during the initial hospitalization period after preterm birth may be linked with benefits for long-term neurodevelopment. However, these intakes, which in our cohort fall below current recommendations, may not act so much as an independent exposure, but rather mediate or reflect the severity of neonatal illness. Future long-term studies in cohorts who have received higher nutrient intakes in line with current guidelines are warranted to either confirm or refute this conclusion.

## Supporting information

S1 Table Executive functioning, attention, and memory in very low birth weight (<1500g) adults. (PDF)

S2 Table Intake of carbohydrates, protein, and fats from birth to 3 weeks, 3 to 6 weeks, and 6 to 9 weeks of age, and IQ, VIQ, and PIQ in young adulthood, in individuals born with very low birth weight (<1500g).^a^ (PDF)

S3 Table Total energy intake and energy intake from human milk from birth to 3 weeks, 3 to 6 weeks, and 6 to 9 weeks of age, and adult IQ, presented separately for those with and without specific neonatal complications and illnesses, in individuals born with very low birth weight (<1500g). (PDF)

## References

[1] Aarnoudse-Moens CSH, Weisglas-Kuperus N, van Goudoever JB, Oosterlaan J. Meta-analysis of neurobehavioral outcomes in very preterm and/or very low birth weight children. Pediatrics. 2009;124(2):717–28. doi: 10.1542/peds.2008-2816 19651588 10.1542/peds.2008-2816

[2] Løhaugen GCC, Gramstad A, Evensen KAI, Martinussen M, Lindqvist S, Indredavik M, et al Cognitive profile in young adults born preterm at very low birthweight. Dev Med Child Neurol. 2010;52(12):1133–8. doi: 10.1111/j.1469-8749.2010.03743.x 21175467 10.1111/j.1469-8749.2010.03743.x

[3] Pyhälä R, Lahti J, Heinonen K, Pesonen A-K, Strang-Karlsson S, Hovi P, et al Neurocognitive abilities in young adults with very low birth weight. Neurology. 2011;77(23):2052–60. doi: 10.1212/WNL.0b013e31823b473e 22146921 10.1212/WNL.0b013e31823b473e

[4] Breeman LD, Jaekel J, Baumann N, Bartmann P, Wolke D. Preterm cognitive function into adulthood. Pediatrics. 2015;136(3):415–23. doi: 10.1542/peds.2015-0608 26260714 10.1542/peds.2015-0608

[5] Ong KK, Kennedy K, Castañeda-Gutiérrez E, Forsyth S, Godfrey KM, Koletzko B, et al Postnatal growth in preterm infants and later health outcomes: A systematic review. Acta Paediatr. 2015;104(10):974–86. doi: 10.1111/apa.13128 26179961 10.1111/apa.13128 PMC5054880

[6] Sammallahti S, Pyhälä R, Lahti M, Lahti J, Pesonen A-K, Heinonen K, et al Infant growth after preterm birth and neurocognitive abilities in young adulthood. J Pediatr. 2014;165(6):1109–15. doi: 10.1016/j.jpeds.2014.08.028 25262301 10.1016/j.jpeds.2014.08.028

[7] Ehrenkranz RA, Das A, Wrage LA, Poindexter BB, Higgins RD, Stoll BJ, et al Early nutrition mediates the influence of severity of illness on extremely low birth weight infants. Pediatr Res. 2011;69(6):522–529. doi: 10.1203/PDR.0b013e318217f4f1 21378596 10.1203/PDR.0b013e318217f4f1 PMC3090495

[8] Isaacs EB, Morley R, Lucas A. Early diet and general cognitive outcome at adolescence in children born at or below 30 weeks gestation. J Pediatr. 2009;155(2):229–34. doi: 10.1016/j.jpeds.2009.02.030 19446846 10.1016/j.jpeds.2009.02.030

[9] Isaacs EB, Gadian DG, Sabatini S, Chong WK, Quinn BT, Fischl BR, et al The effect of early human diet on caudate volumes and IQ. Pediatr Res. 2008;63(3):308–14. doi: 10.1203/PDR.0b013e318163a271 18287970 10.1203/PDR.0b013e318163a271

[10] Isaacs EB, Fischl BR, Quinn BT, Chong WK, Gadian DG, Lucas A. Impact of breast milk on IQ, brain size and white matter development. Pediatr Res. 2010;67(4):357–62. doi: 10.1203/PDR.0b013e3181d026da 20035247 10.1203/PDR.0b013e3181d026da PMC2939272

[11] Brandt I, Sticker EJ, Lentze MJ. Catch-up growth of head circumference of very low birth weight, small for gestational age preterm infants and mental development to adulthood. J Pediatr. 2003;142(5):463–70. doi: 10.1067/mpd.2003.149 12756374 10.1067/mpd.2003.149

[12] Hovi P, Andersson S, Eriksson JG, Järvenpää A-L, Strang-Karlsson S, Mäkitie O, et al Glucose regulation in young adults with very low birth weight. N Engl J Med. 2007;356(20):2053–63. doi: 10.1056/NEJMoa067187 17507704 10.1056/NEJMoa067187

[13] Heinonen K, Pesonen A-K, Lahti J, Pyhälä R, Strang-Karlsson S, Hovi P, et al Self- and parent-rated executive functioning in young adults with very low birth weight. Pediatrics. 2013;131(1):e243–50. doi: 10.1542/peds.2012-0839 23209110 10.1542/peds.2012-0839

[14] Sammallahti S, Lahti M, Pyhälä R, Lahti J, Pesonen A, Heinonen K, et al Infant growth after preterm birth and mental health in young adulthood. PLoS One. 2015;10(9):e0137092 doi: 10.1371/journal.pone.0137092 26327229 10.1371/journal.pone.0137092 PMC4556664

[15] Pyhälä R, Hovi P, Lahti M, Sammallahti S, Lahti J, Heinonen K, et al Very low birth weight, infant growth, and autism-spectrum traits in adulthood. Pediatrics. 2014;134(6):1075–83. doi: 10.1542/peds.2014-1097 25367538 10.1542/peds.2014-1097

[16] Matinolli H-M, Hovi P, Männistö S, Sipola-Leppänen M, Eriksson JG, Mäkitie O, et al Early protein intake is associated with body composition and resting energy expenditure in young adults born with very low birth weight. J Nutr. 2015;145(9):2084–91. doi: 10.3945/jn.115.212415 26180246 10.3945/jn.115.212415

[17] Anderson DM, Williams FH, Merkatz RB, Schulman PK, Kerr DS, Pittard WB 3rd. Length of gestation and nutritional composition of human milk. Am J Clin Nutr. 1983;37(5):810–4. 6846220 10.1093/ajcn/37.5.810

[18] Rönnholm KAR, Sipilä I, Siimes MA. Human milk protein supplementation for the prevention of hypoproteinemia without metabolic imbalance in breast milk-fed, very low-birth-weight infants. J Pediatr. 1982;101(2):243–7. 7097422 10.1016/s0022-3476(82)80133-9

[19] Rönnholm KA, Simell O, Siimes MA. Human milk protein and medium-chain triglyceride oil supplementation of human milk: Plasma amino acids in very low-birth-weight infants. Pediatrics. 1984;74(5):792–9. 6387613

[20] Wechsler D. Wechsler Adult Intelligence Scale 3rd edition (WAIS-III), Finnish version. Helsinki, Finland: Psykologien Kustannus Oy; 2005.

[21] Lezak MD. Neuropsychological assessment. New York: Oxford University Press; 2004.

[22] Rey A. L’examen psychologique dans les cas d’encéphalopathie traumatique Arch Psychol (Geneve). Switzerland: Editions Médecine et Hygiène; 1941;28:215–85.

[23] Reitan RM. Validity of the Trail Making Test as an indicator of organic brain damage. Percept Mot Skills. US: Perceptual & Motor Skills; 1958;8:271–6.

[24] Bohnen N, Jolles J, Twijnstra A. Modification of the Stroop Color Word Test improves differentiation between patients with mild head injury and matched controls. Clin Neuropsychol. 1992;6(2):178–84. 10.1080/13854049208401854 29022450

[25] Conners C. The Conners’ Continuous Performance Test (CPT II). Toronto, Canada: Multi Health Systems; 2004.

[26] National High Blood Pressure Education Program Working Group. Report of the national high blood pressure education program working group on high blood pressure in pregnancy. Am J Obstet Gynecol. 2000;183(1):S1–22. 10920346

[27] Pihkala J, Hakala T, Voutilainen P, Raivio K. Characteristics of recent fetal growth curves in Finland. Duodecim. 1989;105(18):1540–6. 2680445

[28] Northway WH, Rosan RC, Porter DY. Pulmonary disease following respirator therapy of hyaline-membrane disease—Bronchopulmonary Dysplasia. N Engl J Med. 1967; (276):357–68. 5334613 10.1056/NEJM196702162760701

[29] Järvenpää A-L, Granström M-L. The development, social behavior and prognosis of premature infants. [In Finnish]. Duodecim. 1987;103:1238–46. 2458225

[30] Stephens BE, Walden R V, Gargus RA, Tucker R, McKinley L, Mance M, et al First-week protein and energy intakes are associated with 18-month developmental outcomes in extremely low birth weight infants. Pediatrics. 2009;123(5):1337–43. doi: 10.1542/peds.2008-0211 19403500 10.1542/peds.2008-0211

[31] Eleni dit Trolli S, Kermorvant-Duchemin E, Huon C, Bremond-Gignac D, Lapillonne A. Early lipid supply and neurological development at one year in very low birth weight (VLBW) preterm infants. Early Hum Dev. 2012;88:S25–9. 10.1016/j.earlhumdev.2011.12.024 22264437

[32] Brown J, Embleton N, Harding J, McGuire W. Multi-nutrient fortification of human milk for preterm infants. Cochrane Database Syst Rev. 2016; (5):CD000343 doi: 10.1002/14651858.CD000343.pub3 27155888 10.1002/14651858.CD000343.pub3

[33] Tan M, Abernethy L, Cooke R. Improving head growth in preterm infants—a randomised controlled trial II: MRI and developmental outcomes in the first year. Arch Dis Child Fetal Neonatal Ed. 2008;93(5):F342–6. doi: 10.1136/adc.2007.124255 18285378 10.1136/adc.2007.124255

[34] Fenton TR, Premji SS, Al-Wassia H, Sauve RS. Higher versus lower protein intake in formula-fed low birth weight infants. Cochrane Database Syst Rev. 2014; (4):CD003959 doi: 10.1002/14651858.CD003959.pub3 24752987 10.1002/14651858.CD003959.pub3 PMC7104240

[35] Moe-Byrne T, Brown JVE, McGuire W. Glutamine supplementation to prevent morbidity and mortality in preterm infants. Cochrane Database Syst Rev. 2016; (4):CD001457. 10.1002/14651858.CD001457.pub6 PMC7055588 27089158

[36] Bellagamba MP, Carmenati E, D’Ascenzo R, Malatesta M, Spagnoli C, Biagetti C, et al One extra gram of protein to preterm infants from birth to 1800g: A single-blinded randomized clinical trial. J Pediatr Gastroenterol Nutr. 2016;62(6):879–84. doi: 10.1097/MPG.0000000000000989 26418211 10.1097/MPG.0000000000000989

[37] Schulzke SM, Patole SK, Simmer K. Longchain polyunsaturated fatty acid supplementation in preterm infants. Cochrane Database Syst Rev. 2011; (2):CD000375 doi: 10.1002/14651858.CD000375.pub4 27995607 10.1002/14651858.CD000375.pub5 PMC6463838

[38] Collins CT, Gibson RA, Anderson PJ, McPhee AJ, Sullivan TR, Gould JF, et al Neurodevelopmental outcomes at 7 years’ corrected age in preterm infants who were fed high-dose docosahexaenoic acid to term equivalent: A follow-up of a randomised controlled trial. BMJ Open. 2015;5(3):e007314 doi: 10.1136/bmjopen-2014-007314 25787990 10.1136/bmjopen-2014-007314 PMC4368907

[39] Vohr BR, Poindexter BB, Dusick AM, McKinley LT, Higgins RD, Langer JC, et al Persistent beneficial effects of breast milk ingested in the neonatal intensive care unit on outcomes of extremely low birth weight infants at 30 months of age. Pediatrics. 2007;120(4):e953–9. doi: 10.1542/peds.2006-3227 17908750 10.1542/peds.2006-3227

[40] Rozé J-C, Darmaun D, Boquien C-Y, Flamant C, Picaud J-C, Savagner C, et al The apparent breastfeeding paradox in very preterm infants: Relationship between breast feeding, early weight gain and neurodevelopment based on results from two cohorts, EPIPAGE and LIFT. BMJ Open. 2012;2:e000834 doi: 10.1136/bmjopen-2012-000834 22492388 10.1136/bmjopen-2012-000834 PMC3323805

[41] Gibertoni D, Corvaglia L, Vandini S, Rucci P, Savini S, Alessandroni R, et al Positive effect of human milk feeding during NICU hospitalization on 24 month neurodevelopment of very low birth weight infants: An Italian cohort study. PLoS One. 2015;10(1):e0116552 doi: 10.1371/journal.pone.0116552 25590630 10.1371/journal.pone.0116552 PMC4295863

[42] Johnson S, Wolke D, Hennessy E, Marlow N. Educational outcomes in extremely preterm children: Neuropsychological correlates and predictors of attainment. Dev Neuropsychol. 2011;36(1):74–95. doi: 10.1080/87565641.2011.540541 21253992 10.1080/87565641.2011.540541

[43] Belfort MB, Anderson PJ, Nowak VA, Lee KJ, Molesworth C, Thompson DK, et al Breast milk feeding, brain development, and neurocognitive outcomes: A 7-year longitudinal study in infants born at less than 30 weeks’ gestation. J Pediatr. 2016;177:133–9. doi: 10.1016/j.jpeds.2016.06.045 27480198 10.1016/j.jpeds.2016.06.045 PMC5037020

[44] O’Connor DL, Jacobs J, Hall R, Adamkin D, Auestad N, Castillo M, et al Growth and development of premature infants fed predominantly human milk, predominantly premature infant formula, or a combination of human milk and premature formula. J Pediatr Gastroenterol Nutr. 2003;37:437–46. 14508214 10.1097/00005176-200310000-00008

[45] Furman L, Wilson-Costello D, Friedman H, Taylor HG, Minich N, Hack M. The effect of neonatal maternal milk feeding on the neurodevelopmental outcome of very low birth weight infants. J Dev Behav Pediatr. 2004;25(4):247–53. 15308925 10.1097/00004703-200408000-00004

[46] Smith MM, Durkin M, Hinton VJ, Bellinger D, Kuhn L. Influence of breastfeeding on cognitive outcomes at age 6–8 years: Follow-up of very low birth weight infants. Am J Epidemiol. 2003;158(11):1075–82. 14630603 10.1093/aje/kwg257

[47] Lucas A, Morley R, Cole T, Gore S. A randomized multicenter study of human-milk versus formula and later development in preterm infants. Arch Dis Child. 1994;70:F141–146. 10.1136/fn.70.2.f141 PMC1061016 8154907

[48] Lucas A, Morley R, Cole TJ. Randomised trial of early diet in preterm babies and later intelligence quotient. BMJ. 1998;317(7171):1481–7. 9831573 10.1136/bmj.317.7171.1481 PMC28727

[49] Hoenig JMJ, Heisey DDM. The abuse of power. Am Stat. 2001;55(1):19–24.

[50] Patel RM, Kandefer S, Walsh MC, Bell EF, Carlo WA, Laptook AR, et al Causes and timing of death in extremely premature infants from 2000 through 2011. N Engl J Med. 2015;372:331–40. doi: 10.1056/NEJMoa1403489 25607427 10.1056/NEJMoa1403489 PMC4349362

[51] Agostoni C, Buonocore G, Carnielli VP, De Curtis M, Darmaun D, Decsi T, et al Enteral nutrient supply for preterm infants: Commentary from the European Society of Paediatric Gastroenterology, Hepatology and Nutrition Committee on Nutrition. J Pediatr Gastroenterol Nutr. 2010;50(1):85–91. doi: 10.1097/MPG.0b013e3181adaee0 19881390 10.1097/MPG.0b013e3181adaee0

