# Supplementary material for: Nutrition after preterm birth and adult neurocognitive outcomes
Source: PLoS One. 2017 Sep 28;12(9):e0185632. doi: 10.1371/journal.pone.0185632 (PMC5619810; doi:10.1371/journal.pone.0185632)
Supplement: S1 Table — (PDF) [file pone.0185632.s001.pdf]

S1 Table. Executive functioning, attention, and memory in very low birth weight (<1500g) adults.

Neurocognitive test score	*Component loading* [a]				*M (SD)*	Participants [b]
	1. Verbal flexibility	*2. Visual memory*	*3. Visual flexibility*	*4. Impul-sivity*		
**Verbal Fluency Task**						
Phonetic raw test score mean	0.83	0.16	0.18	-0.01	12 (4.3)	86
Category raw test score mean	0.87	0.08	-0.12	0.05	19 (4.9)	86
**Rey-Osterrieth Complex Figure Test**						
Copy, raw test score	0.15	0.55	0.14	-0.08	34 (3.3)	86
Immediate recall, raw test score	0.05	0.95	0.02	0.05	22 (7.4)	86
Delayed recall, raw test score	0.01	0.96	0.05	0.04	22 (7.2)	86
**Trail Making Test** [c]						
Part A, seconds	0.08	0.02	0.87	0.05	38 (15)	86
Part B, seconds	0.29	0.24	0.80	-0.04	78 (39)	86
**Stroop Test** [c]						
Baseline task, seconds	0.70	0.03	0.42	0.07	78 (18)	86
Interference task, seconds	0.65	0.02	0.41	-0.04	133 (37)	85
**Conners' Continuous Performance Test** [c]						
Number of commission errors	0.09	-0.004	0.09	-0.92	11 (7.1)	85
Hit reaction time, milliseconds	0.15	-0.01	0.12	0.90	350 (54)	85

[a] Rotated Component Matrix; using principal component analysis all measures of executive function, attention, and visual memory (in SD units) were re-organized into 4 components, named Verbal flexibility (1), Visual memory (2), Visual flexibility (3) and Impulsivity (4).

[b] 84 participants had data available on all these tests of executive functioning, attention, and memory, and were thus included in the principal component analysis which yielded the components that we used as final outcome variables in relation to nutrition.

[c] For the principal component analysis, test SD scores were multiplied by -1 so that higher scores reflect better performance.

Abbreviations: M: mean; Participants: number of participants for whom data were available; SD: standard deviation
