# Supplementary material for: Nutrition after preterm birth and adult neurocognitive outcomes
Source: PLoS One. 2017 Sep 28;12(9):e0185632. doi: 10.1371/journal.pone.0185632 (PMC5619810; doi:10.1371/journal.pone.0185632)
Supplement: S2 Table — (PDF) [file pone.0185632.s002.pdf]

S2 Table. Intake of carbohydrates, protein, and fats from birth to 3 weeks, 3 to 6 weeks, and 6 to 9 weeks of age, and IQ, VIQ, and PIQ in young adulthood, in individuals born with very low birth weight (<1500g).[a]

	IQ		*VIQ*		*PIQ*	
	Effect size	*(95% CI)*	*Effect size*	*(95% CI)*	*Effect size*	*(95% CI)*
**Carbohydrates**						
Birth to 3 weeks	-0.01	(-0.16, 0.15)	-0.10	(-0.25, 0.05)	0.07	(-0.09, 0.23)
3-6 weeks	0.18	(0.02, 0.35)	0.18	(0.01, 0.34) [b]	0.14	(-0.04, 0.31)
6-9 weeks	0.08	(-0.09, 0.25)	0.11	(-0.05, 0.28)	0.04	(-0.14, 0.22)
**Protein**						
Birth to 3 weeks	0.33	(-0.22, 0.87)	-0.20	(-0.74, 0.34)	0.73	(0.18, 1.29) [b]
3-6 weeks	0.39	(-0.11, 0.90)	0.19	(-0.30, 0.69)	0.47	(-0.06, 1.00)
6-9 weeks	-0.13	(-0.59, 0.33)	-0.26	(-0.70, 0.19)	-0.04	(-0.52, 0.44)
**Fats**						
Birth to 3 weeks	0.16	(-0.02, 0.34)	0.03	(-0.15, 0.21)	0.25	(0.07, 0.44) [b]
3-6 weeks	0.34	(0.14, 0.54) [bc]	0.25	(0.05, 0.45) [bc]	0.35	(0.14, 0.56) [b]
6-9 weeks	0.14	(-0.09, 0.36)	0.13	(-0.09, 0.35)	0.11	(-0.13, 0.35)

In all analyses, we adjusted for gestational age, sex, birth weight standard deviation score, and age at follow-up (Model 1).

[a] The number of participants in each analysis varied according to data availability. At 0-3 weeks, 86 participants; at 3-6 weeks, 82 participants; and at 6-9 weeks, 79 participants had data available on carbohydrate, protein, and fat intakes and were included in the analyses (Model 1-2). In Model 3, we had to further exclude two people because of missing data on neonatal complications.

[b] p-value <0.05 in Model II (adjusted for Model I factors, parental education, maternal smoking during pregnancy, and preeclampsia).

[c] p-value <0.05 in Model III (when adjusting for Model I factors and neonatal complications, including septicemia, bronchopulmonary dysplasia, patent ductus arteriosus, blood exchange transfusion, duration of ventilator treatment, and intraventricular hemorrhage).

Abbreviations: Carbohydrates: mean carbohydrate intake, g/kg/day; CI: Confidence interval; Effect size: change in IQ, VIQ, or PIQ, in standard deviation units, for each g/kg/day increase in carbohydrate, protein, and fat intake; Fats: mean fat intake, g/kg/day; g: gram; kcal: kilocalories; IQ: full-scale intelligence quotient; PIQ: performance intelligence quotient; Protein: mean protein intake, g/kg/day; VIQ: verbal intelligence quotient
