# Supplementary material for: Nutrition after preterm birth and adult neurocognitive outcomes
Source: PLoS One. 2017 Sep 28;12(9):e0185632. doi: 10.1371/journal.pone.0185632 (PMC5619810; doi:10.1371/journal.pone.0185632)
Supplement: S3 Table — (PDF) [file pone.0185632.s003.pdf]

S3 Table. Total energy intake and energy intake from human milk from birth to 3 weeks, 3 to 6 weeks, and 6 to 9 weeks of age, and adult IQ, presented separately for those with and without specific neonatal complications and illnesses, in individuals born with very low birth weight (<1500g).

**Neonatal complications and illnesses**	**N**	**Total energy intake, kcal/kg/day**			**Energy from human milk, kcal/kg/day**			**IQ**
		Birth to 3 weeks	*3-6 weeks*	*6-9 weeks*	*Birth to 3 weeks*	*3-6 weeks*	*6-9 weeks*	
		M (SD)	M (SD)	M (SD)	M (SD)	M (SD)	M (SD)	M (SD)
Duration of ventilator treatment, *p*		*<0.001*	*0.18*	*0.79*	*<0.001*	*0.001*	*0.23*	*0.13*
None	22	101 (11)	123 (8)	129 (17)	87 (21)	118 (15)	116 (18)	105 (13)
1-7 days	24	101 (16)	123 (11)	124 (9)	89 (18)	116 (12)	112 (25)	103 (14)
8-14 days	13	94 (11)	123 (16)	125 (13)	77 (16)	105 (21)	102 (26)	107 (15)
15-28 days	8	84 (13)	116 (16)	124 (15)	65 (28)	104 (19)	102 (36)	96 (21)
More than 28 days	17	81 (19)	114 (15)	126 (12)	55 (23)	94 (23)	101 (23)	95 (16)
Septicemia, *p*		*0.23*	*0.61*	*0.82*	*0.20*	*0.27*	*0.30*	*0.12*
No	76	95 (16)	121 (13)	126 (13)	79 (24)	110 (19)	108 (25)	103 (15)
Yes	8	87 (19)	118 (17)	125 (17)	65 (30)	102 (19)	117 (19)	93 (21)
Bronchopulmonary dysplasia, *p*		*0.001*	*0.29*	*0.97*	*<0.001*	*<0.001*	*0.37*	*0.27*
No	62	98 (15)	122 (10)	126 (13)	84 (21)	115 (16)	110 (25)	103 (16)
Yes	22	84 (18)	117 (18)	126 (13)	60 (25)	95 (22)	106 (23)	99 (15)
Received indomethacin, *p*		*0.001*	*0.02*	*0.53*	*<0.001*	*0.001*	*0.18*	*0.18*
No	55	99 (14)	123 (12)	126 (14)	85 (21)	115 (15)	111 (23)	103 (15)
Yes	29	86 (18)	116 (14)	124 (11)	64 (24)	99 (22)	103 (26)	99 (16)
Blood exchange transfusion, *p*		*0.80*	*0.67*	*0.57*	*0.82*	*0.33*	*0.03*	*0.83*
No	70	94 (17)	121 (13)	126 (13)	77 (25)	109 (20)	107 (25)	102 (14)
Yes	14	93 (15)	119 (11)	124 (13)	79 (24)	115 (17)	119 (10)	102 (20)
Intraventricular hemorrhage, [a] *p*		*0.02*	*0.005*	*0.03*	*0.01*	*0.001*	*0.40*	*<0.001*
None	50	96 (16)	122 (11)	128 (14)	79 (25)	112 (18)	107 (26)	106 (12)
Grade I or II	10	87 (16)	113 (16)	123 (6)	68 (20)	93 (23)	108 (21)	92 (14)
Grade III or IV	4	78 (9)	106 (10)	108 (5)	47 (21)	86 (24)	85 (36)	88 (24)

[a] Those who underwent cerebral ultrasound (n=64) did not differ from those who did not undergo ultrasound examination (n=22) in total energy intakes, energy intakes from human milk, or IQ (p-values>0.12).

Abbreviations: IQ: Full-scale intelligence quotient; g: gram; kcal: kilocalorie; M: mean (of total energy intake / energy intake from human milk / IQ score within each group); N: number of participants (in each group); *p*: p-value for difference between groups (for duration of ventilation treatment, analysis of variance was performed, and for other dichotomized complications, t-tests were performed: for these analyses, outcomes were square transformed to attain normality, however means are given as non-transformed values); SD: standard deviation.
